# Covid-19, child and adolescent mental health – Croatian (in)experience

**DOI:** 10.1017/ipm.2020.55

**Published:** 2020-05-21

**Authors:** Tomislav Franic, Katarina Dodig-Curkovic

**Affiliations:** 1 Clinical Hospital Centre Split, Medical School, University of Split, Split, Croatia; 2 Clinical Hospital Centre Osijek, Medical School, University of Osijek, Osijek, Croatia

**Keywords:** Child and adolescent mental health, corona, Covid-19, Croatia

## Abstract

The Covid-19 pandemic has caused unseen socio-economic changes all over the world, where enormous efforts are being made to preserve lives and maintain functional health systems. A secondary concern is to mitigate the severe economic consequences of the crisis. Different approaches have been adopted with varying outcomes and experiences. But regardless of the different approaches taken, one thing is common for all societies during this pandemic: fear and anxiety. This fear extends from concerns about the present situation, for the health and well-being of family members and loved ones from Covid-19 infection, to fears relating to how long the crisis will last, to the potential economic consequences of the pandemic (perhaps not seen in our lifetimes) and the ultimate fear of future uncertainty. Across the world, health systems are being faced with unprecedented challenges. At their core, these challenges are the same: how to beat Covid-19. Certainly, there are differences in how individual systems are organized and how they address the main issues arising from the pandemic while not forgetting the ongoing healthcare needs of the general population. In this paper, we share some perspectives from Croatia regarding Child and Adolescent Mental Health services (CAMHs) in these extraordinary circumstances. We give our personal insights on deficiencies in Child and Adolescent Mental Health Services prior to the arrival of Covid-19, which have contributed to difficulties in mitigating and managing the ongoing crisis.

The first case of Covid-19 in Croatia was confirmed on 25 February 2020. Epidemiologically, it was traced to Milan, and to the Atalanta vs Valencia Champions League football match. This was later proclaimed to be a ‘biological bomb’ which brought infection to Bergamo, probably the worst affected city in the world. A subsequent cluster was also traced to Italy. At the time of writing this paper, Covid-19 infection has spread across all counties in Croatia with a total of 1832 cases, 39 deaths and 615 recovered patients. The mean age of all those infected is 50, and the majority of deaths were cases with comorbidities in older age. The youngest victim was 55 and the youngest infected person is a 4-month-old baby. These numbers, in the context of the time period and overall population, are very good. Croatia has had 446 cases and 9 deaths per million, placing it in fourth place (with Poland) in the EU, behind Slovakia, Latvia and Bulgaria. The epidemiological curve was sharp (20–30% daily rise) for a period of 4–5 days only and is now almost flat with several days of reductions in the number of new cases. Up to now a maximum of 39 ventilators (of the total of 800 available) were required daily (Worldometer, 2020).

The pandemic has caused unseen socio-economic changes in Croatia. Having witnessed the outbreak in Italy, Croatia had time to introduce counter measures. These counter measures were stepwise from the quarantining of passengers from high-risk regions to full lockdown. All of this happened in less than 3 weeks. The speed of the response may indeed be a legacy of our history since the quarantine is a Croatian invention. Throughout its history, Dubrovnik was ravaged by numerous diseases, with leprosy and plague posing the largest threats to public health. In Venice, all ships arriving from infected ports were required to sit at anchor for 40 days (quaranta giorni) before landing, a practice that eventually became known as quarantine (derived from ‘quarantino’, the Italian word for a 40-day period). Unlike Venice, Dubrovnik chose a different approach, being the first Mediterranean port to sequester people, animals and merchandise coming from infected areas by sea or land, keeping them separate from the healthy population, while Venice stopped all ships and trade, halting life in the city. In 1377, Dubrovnik issued a decree according to which ‘risky’ people and goods needed to spend 40 days at one of the nearby islands before entering the city. It was the first such decision in the world and quarantine is now widely considered a Dubrovnik invention (Henry, 2013). Furthermore, Andrija Stampar, the ‘father of public health and prevention’ and founder of the World Health Organisation (WHO), came from Croatia: ‘All our efforts will fail until everybody enjoys the benefits of hygienic culture’ (Brown, 2006).

Currently in Croatia all citizens who are not classified as essential workers are required to remain at home. They are not legally forbidden to go out, but it is strongly recommended not to do so. All public events and gatherings of more than five persons are prohibited and museums, theatres, cinemas, libraries and reading rooms are closed. Exhibitions, shows and fairs, concerts, weddings and religious ceremonies are suspended. Most retail outlets are closed except for those selling food and hygiene items, pharmacies, gas stations, baby equipment and pet food. Play-grounds and public squares are closed and are under police or civil protection task-force supervision to prevent gathering and to supervise physical distancing. Travelling between municipalities is allowed only under specific circumstances and with appropriate e-passes. The crossing of all borders is prohibited or restricted for all Croatian citizens. Only emergency and front-line essential services are allowed to work onsite and face to face. Everything else has either been suspended or transferred to working from home. Even schools are online with additional backup on several national TV channels and all materials made available on YouTube. This has resulted in increased self-isolation and the cutting off of the majority of face to face contact. It is also recommended not to visit older relatives unless for the purpose of delivering food and other necessary supplies.

Other countries took different approaches resulting in different outcomes and experiences, but regardless of the measures taken, one thing is common for all nations: fear and anxiety. People have many fears relating to the present situation: they fear for the health and well-being of family and loved ones regarding covid-19 infection; they fear the length of time the pandemic will last; they fear the economic fallout from the crisis (perhaps unlike anything seen in our lifetimes) and they fear the uncertainty of the future. All these factors are related to the mental well-being of the population, mental health care and mental health outcomes.

In addition to the numerous changes outlined above, health professionals are faced with unprecedented challenges. These challenges, although manifold, boil down to the same thing: how to beat Covid-19 (virology, epidemiology, infectiology, ICU staff and many others). The differences are found in how systems are organized and in how they address the main issue while, at the same time, maintaining the healthcare needs of the general population. Mental health care and Child and Adolescent Mental Health (CAMH) are fundamental, not only because of ongoing care needs, but also due to the probable psychological consequences of the pandemic. It is unquestionable that such sudden and unprecedented change will have some impact on the future psychological status of individuals and of society as a whole. Of course, it is impossible to predict the nature of such change. Unfortunately, it is anticipated that this will be a traumatic process. Needless to say, Croatia has some experience of trauma. It is not that long since it was engaged in a war which had a deep impact not only on those soldiers directly exposed to the fighting, but also on their offspring in the form of transgenerational transfer (Franić *et al.* 2012). This might direct CAMH professionals not only toward the mental health needs of children and adolescents, but also of parents caused by the Covid-19 crisis to address possible mental health issues.

The organization and training of CAMH services in Croatia are detailed elsewhere (Signorini *et al*. 2017; Russet *et al.* 2019). In short, they are insufficient for the approximately 900,000 children and adolescents up to age of 19 in Croatia. Care is free of charge as it is for adult mental health patients. The overall health system in Croatia is publicly funded and free of charge for all, based on fixed contributions from the salaries of all employees. Only a small number of private health facilities exist, mainly in highly profitable services such as plastic surgery, ophthalmology and dental medicine. CAMH services are affiliated mainly with four university hospitals, three of which have inpatient units. There is one standalone Psychiatric Hospital for Children and Youth in Zagreb (PHCY).

There are no community-based psychiatry outpatient services. They are instead affiliated to five hospitals and two additional CAMH outpatient centres. Of course, there are some individual practices (public and private) across the country, but again not enough. According to the draft Strategic Plan of CAMH development in Republic of Croatia 2019–2023, the target number of CAMH psychiatrists is 120. According to the same source, at present the total number of beds in the whole country is 66 with an additional 100 places in daily accommodation. The number of CAMH consultants is 44 with 22 trainees. To put this into context, the number of adult psychiatrists, adjusted according to the number of citizens and prevalence of mental disorders is 150–200, which is three to four times more than in CAMHs.

In short, we faced the Covid-19 crisis with already insufficient resources. To compound this situation, many health facilities have been intentionally sacrificed in the re-organization necessitated by the virus. The Croatian approach is to have separate Covid-19 hospitals which has resulted in the reduction of all elective so-called cold programmes. Both adult and CAMHS psychiatric services are regarded as cold. Furthermore, many wards were removed reducing existing capacities. Since the capacity of adult psychiatry is large enough, somehow, they compensate for this. For CAMHs, it was a completely different story. The services in the University Hospital in Split are completely shut down and the University Hospital in Osijek has lost half of its capacity to adult psychiatry. This was complicated further by that we had a virus outbreak in the biggest, standalone PHCY and patients were discharged or removed to adult wards. If that was not enough, the University Hospital in Zagreb was lost due to an earthquake in the city on 22nd March, the biggest since 1880. Thousands of buildings were damaged, including numerous hospitals. In addition, people left their homes for 2 days breaking physical distance and isolation recommendations.

The catalogue of these events has been described as our Pearl Harbour, but we as professionals have somehow managed to regroup and jointly our CAMH societies wrote and published guidelines regarding CAMH and Covid-19 on the website of *European Child and Adolescent Psychiatry* (ECAP). This includes a range of advice from practical guidelines on how to manage possible Covid-19 infected children, advice to affected families to recommendations on how to manage with anxiety and other possible consequences of the pandemic in the general population of children and adolescents. Also, during this period, we had lot of media exposure on the topic of mental health, and finally on an individual level we managed to introduce new methods not used before such as telepsychiatry and videoconferencing. The response to such innovation has been surprisingly good. As a service, we had little availability (expect for emergencies) for several weeks. People who were desperate in seeking help for their children and adolescents were willing to accept help on this basis. Of course, it was biased by the fact that availability and contact were on a voluntary basis. It is not clear according to Croatian law whether we are allowed to discharge such proactive outreach, but since extraordinary times deserve extraordinary measures we are working on the edge of normal ethics considerations and GDPR rules. As we did not previously have such IT practices, we did not have consent for this approach. We have put ourselves at some risk for the sake of our patients who were completely lacking any professional support. So far, feedback has been positive and we have not been subject to litigation. Things are slowly going back to normal or as close to normal as possible.

In Croatia, CAMH professionals have been confronted not only by significant obstacles caused by the Covid-19 pandemic but also by the erratic and catastrophic organizational changes which have ensued. These did not consider CAMH services in Croatia as equal, despite the number of young and vulnerable patients requiring ongoing services. We are now on the journey of returning to the level of care as it previously was but with different means and in unusual times. Fortunately, the initial impressions suggest that the children are much more resilient and less anxious about the changes than parents or professionals.

To conclude, this crisis has facilitated new forms of professional help and treatment for which we were not prepared, but we have been forced to improvise. Our first impressions have shown that we, as well our patients, have adapted positively to such novel clinical techniques. Bearing in mind the pre-existing deficiencies in CAMHs and in the healthcare system overall, such new techniques may indeed be helpful when the crisis has passed. When that time comes, we expect to see an increased number of children with psychological consequences related to the Covid-19 crisis. We need to prepare now to ensure that the best care and treatment will be delivered at this time.
